# Intestinal Metastasis of Choriocarcinoma: A Case Report of a Rare Cause of Gastrointestinal Bleeding

**DOI:** 10.7759/cureus.82220

**Published:** 2025-04-14

**Authors:** Kevin Winston, Edo Rezaprasga, Syahidatul Wafa

**Affiliations:** 1 Department of Internal Medicine, Cipto Mangunkusumo National General Hospital, Jakarta, IDN; 2 Division of Endocrinology and Metabolism, Department of Internal Medicine, Cipto Mangunkusumo National General Hospital, Jakarta, IDN

**Keywords:** choriocarcinoma, gastrointestinal bleeding, gastrointestinal metastasis, gestational trophoblastic disease, hematochezia

## Abstract

Choriocarcinoma is widely known as an aggressive gestational trophoblastic disease characterized by rapid proliferation and early hematogenous spread. Gastrointestinal metastases, particularly to the small intestine, are rare but can lead to life-threatening complications such as ulceration, perforation, and massive bleeding. This case report describes intestinal metastasis of choriocarcinoma in a 30-year-old patient presenting with chronic hematochezia, progressive pallor, unintentional weight loss over three months, and left lower abdominal pain. Previously, the patient had a molar pregnancy, which was removed via dilation and curettage procedure, but no subsequent follow-up and examinations were conducted post-operation. Physical examination revealed conjunctival pallor and a palpable mass in the left lower quadrant, with rectal examination confirming active bleeding. Chest X-ray revealed multiple pulmonary nodules, and ultrasound revealed abdomen liver metastasis and irregular mass in the lower abdomen. The same mass was confirmed by CT scan of the abdomen. During hospitalization, the patient experienced recurrent GI bleeding, necessitating multiple blood transfusions. Colonoscopy identified an actively bleeding mass in the terminal ileum, and an abdominal CT scan confirmed a large abdominal mass. Extremely elevated levels of hCG (>1,000,000 mIU/mL) supported the diagnosis of metastatic gestational trophoblastic cancer with subsequent histopathological analysis confirmed metastatic choriocarcinoma. Given the persistent hemorrhage, surgical intervention was performed, but the patient developed postoperative sepsis and succumbed to the illness. Although choriocarcinoma is highly treatable with chemotherapy, atypical metastases to the gastrointestinal tract can complicate diagnosis and management, leading to severe complications. This case emphasizes the importance of considering choriocarcinoma in the differential diagnosis of unexplained GI bleeding in patients with a history of gestational trophoblastic disease.

## Introduction

Choriocarcinoma is a rare but highly aggressive distinct entity of gestational trophoblastic disease (GTD) that originates from the trophoblastic cells [1-3]. Importantly, choriocarcinoma is characterized by rapid proliferation and early hematogenous spread, most commonly to the lungs, liver, and brain [4]. Furthermore, choriocarcinoma is associated with complete or incomplete molar pregnancy (hydatidiform mole) [5,6]. Thus, follow-up of molar pregnancy in patients is important due to potential malignant complications. As choriocarcinoma is distinguished by its ability to produce high levels of human chorionic gonadotropin (hCG), periodic monitoring of hCG can be used to determine relapse or aid in diagnosis [1-3]. Interestingly, despite its aggressive nature, choriocarcinoma is highly sensitive to chemotherapy, and with early detection and appropriate treatment, the prognosis is generally favorable [1-3,7].

Symptoms of choriocarcinoma may include abnormal uterine bleeding with abdominal pain and irregular periods. However, not all patients may present with these symptoms, and some patients may even be asymptomatic [8]. Metastasis may produce additional symptoms depending on locations [9,10]. For example, CNS metastasis may cause seizure, persistent headache, or dizziness. Meanwhile, hemoptysis and dyspnea may be seen in pulmonary metastasis [9,10].

According to guidelines from the Royal College of Obstetricians and Gynaecologists, any woman with persistent vaginal bleeding after a pregnancy event is at risk of gestational trophoblastic neoplasia (GTN), such as choriocarcinoma, and should therefore be investigated for GTN [9]. The guideline recommends a urinary hCG test, as it can aid in the diagnosis of GTN. A normal hCG level eight weeks after molar pregnancy removal has a high likelihood of excluding GTN, such as choriocarcinoma [9,11]. In addition, the guideline recommends that all women with a molar pregnancy be referred for follow-up at a specialized GTD center [9].

As stated, the site of metastasis for choriocarcinoma commonly involves the lungs, liver, and brain [12-14]. However, in extremely rare cases, choriocarcinoma may metastasize to the intestines, and when choriocarcinoma metastasizes to the intestines, it can lead to severe complications such as life-threatening gastrointestinal (GI) bleeding [12,13]. Furthermore, the presentation of metastatic choriocarcinoma in the intestines can mimic other GI pathologies, often leading to delayed diagnosis.

This report describes the case of a patient with previously operated molar pregnancy through dilation and curettage who had inadequate follow-up post-operation and presented to our center due to chronic hematochezia from small intestine metastasis of choriocarcinoma. This case highlights the rare but serious complication of intestinal metastasis in choriocarcinoma, emphasizing the challenges in the diagnosis and management of such patients.

## Case presentation

A 30-year-old female patient presented with the chief complaint of chronic hematochezia that began one month prior to admission, with increasing frequency. The patient also reported generalized weakness and pallor. Additionally, the patient experienced constant left lower abdominal pain for the past three weeks. The pain was not influenced by changes in position or physical activity and was non-radiating. The patient had not undergone any prior diagnostic imaging such as a CT scan or MRI.

Over the preceding three months, the patient reported unintentional weight loss amounting to 10 kg. The patient denied having symptoms such as cough, shortness of breath, recurrent fever, joint pain, recurrent mouth ulcers, red eyes, or back pain. There was no prior history of hemorrhoids or GI bleeding. The patient denied using aspirin or anticoagulants. Additionally, there was no family history of cancer or inflammatory bowel disease.

The patient provided a history of a molar pregnancy of approximately eight months prior to presentation, which was diagnosed on ultrasound in a different hospital. The patient experienced amenorrhea three months before diagnosis but reported vaginal bleeding two months prior to diagnosis. After the diagnosis, the patient underwent dilation and curettage procedure to remove the molar pregnancy, but subsequent follow-up imaging and post-surgical hCG level monitoring were not conducted due to insurance limitations. Furthermore, no pathological examination was conducted after the surgical curettage. Since there were no symptoms after the surgery, the patient did not return to the hospital for examinations.

On physical examination, vital signs were within normal limits: blood pressure 116/71 mmHg, heart rate 133 beats per minute, respiratory rate 20 breaths per minute, temperature 37°C, and oxygen saturation (SpO_2_) 98% on room air. BMI of the patient was 22.4 kg/m^2^. Conjunctival pallor was observed. The most significant finding on abdominal examination was the presence of a palpable mass in the lower left quadrant. Rectal examination did not reveal any masses but was notable for bleeding.

**Figure 1:**
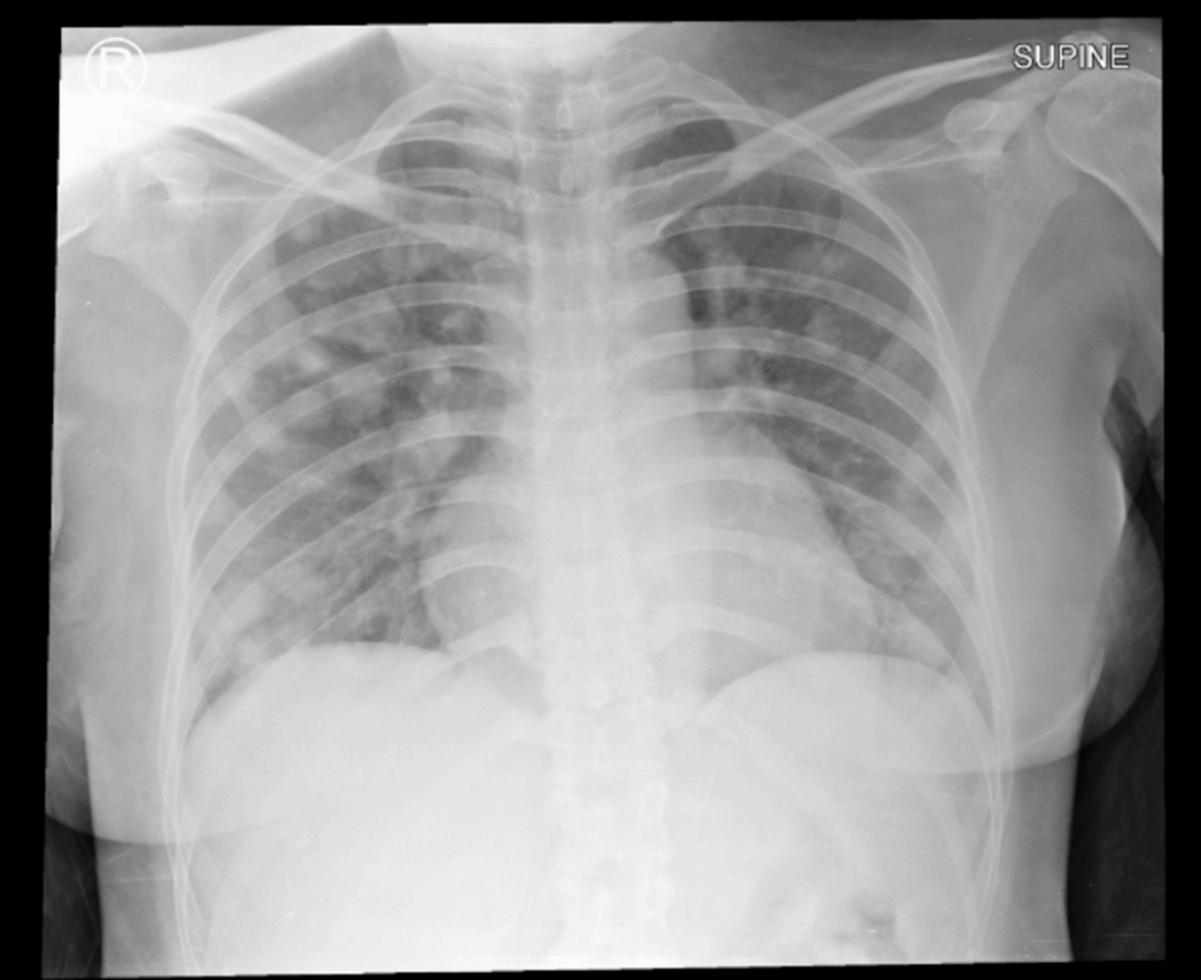
Chest X-ray of the patient showing multiple lung nodules.

**Figure 2:**
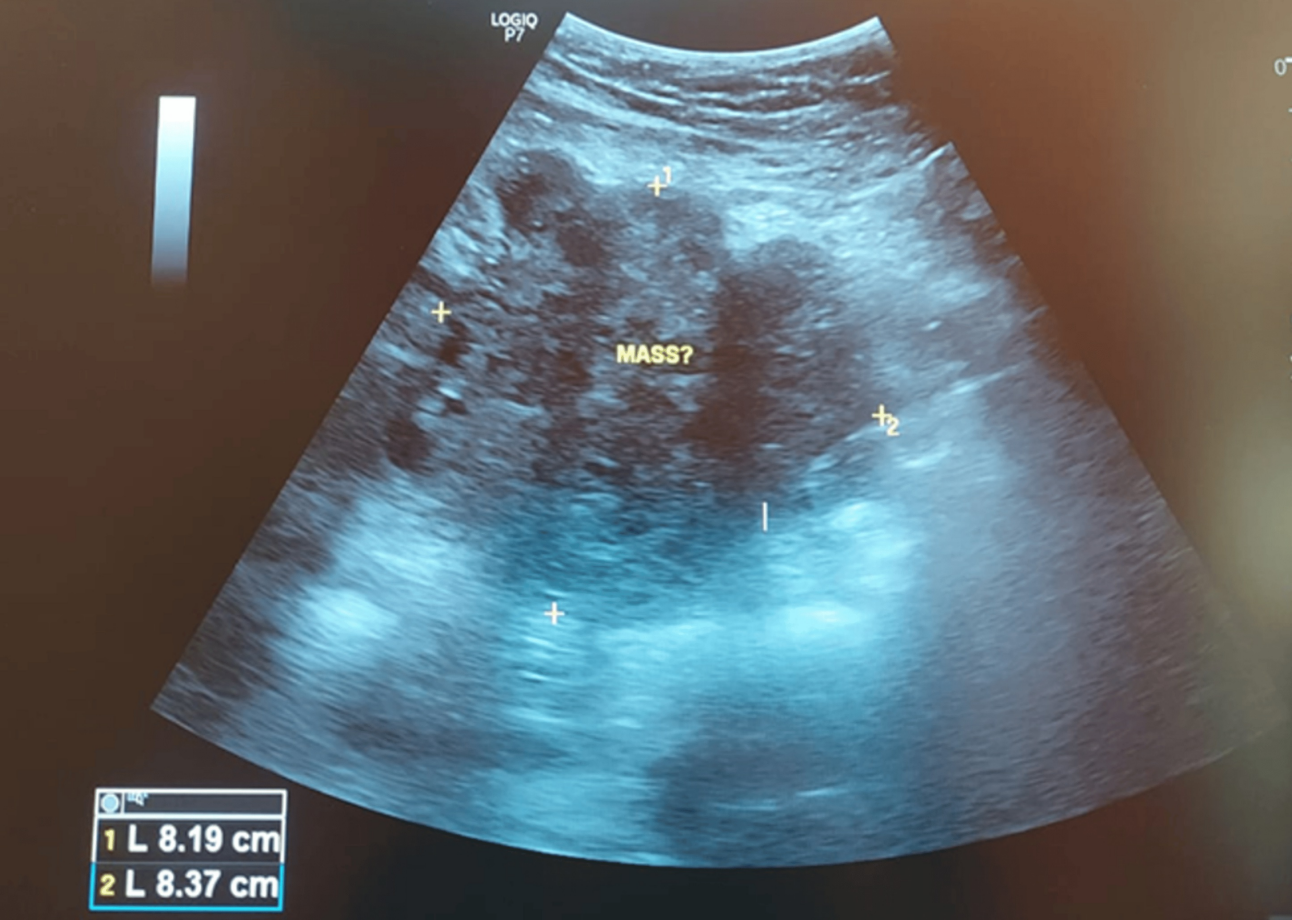
Ultrasound showing a mass on the lower left abdomen.

Initial laboratory investigations revealed a marked decrease in hemoglobin levels, with a value of 5.7 g/dL, indicating severe anemia. Chest X-ray revealed multiple nodular opacities of varying sizes scattered throughout both lungs, predominantly in the right lung (Figure 1). Bedside ultrasound revealed multiple liver nodules and an irregular mass on the lower left abdomen was observed with an estimated size of 8.19 cm x 8.37 cm (Figure 2). Based on the International Federation of Gynaecology and Obstetrics (FIGO) 2000 Prognostic Scoring System for GTN, the patient had a score of 16, which indicated a high risk for mortality [15].

During hospitalization, the patient experienced multiple episodes of GI bleeding, resulting in low hemoglobin. Thus, the patient was treated with multiple packed red cell transfusions, with a target hemoglobin level of 10 g/dL.

The patient was also prepared for a colonoscopy to diagnose a suspected mass in the lower GI tract using whole bowel irrigation. However, before the colonoscopy preparation was completed, the patient experienced multiple severe episodes of hematochezia, leading to hemorrhagic shock due to the administration of polyethylene glycol induced profuse bleeding, causing the patient to enter hemorrhagic shock once again. The condition was stabilized through additional packed red cell transfusions.

**Figure 3:**
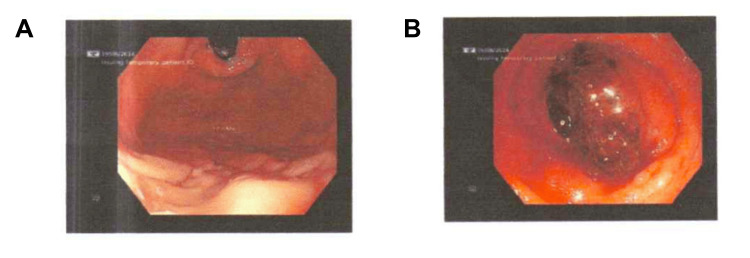
(A) Endoscopy with no abnormal bleeding in the stomach. (B) Colonoscopy showing a mass in the terminal ileum with active bleeding.

An upper GI endoscopy was also conducted, which showed no bleeding from the upper GI tract (Figure 3A). The patient also underwent a colonoscopy, which revealed an actively protruding bleeding mass located in the terminal ileum (Figure 3B). A biopsy was conducted for pathological examination.

**Figure 4:**
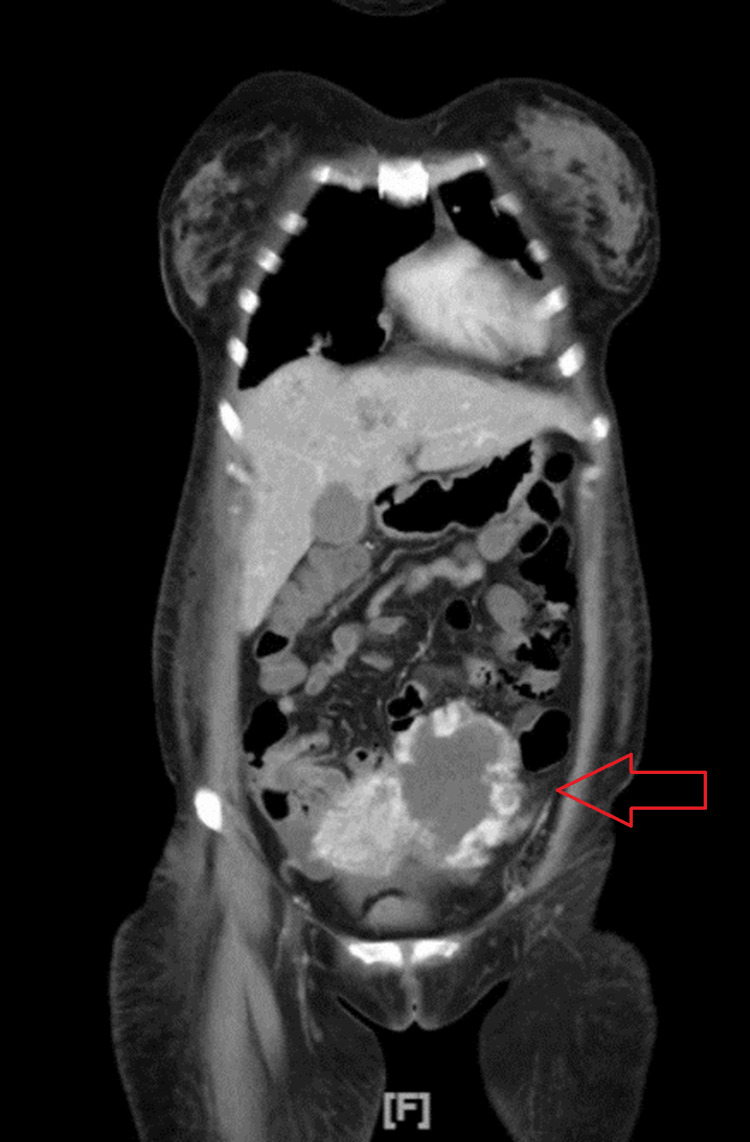
Result of abdominal CT scan showing a mass on the left lower abdomen (arrow).

A contrast-enhanced abdominal CT scan was subsequently performed, revealing a mass in the lower left abdomen with a size of 4.1 x 4.9 x 6.7 cm (Figure 4). Furthermore, ileocolic-ileocecal intussusception was observed with a hypervascular mass in the ileum. Elevation of CA-125 (281.7 U/mL) along with extremely elevated hCG (> 1,000,000 mIU/mL) suggested gestational trophoblastic cancer.

Due to persistent bleeding and the need for repeated transfusions, surgical intervention was deemed necessary. Previously, arterial embolization to stop the bleeding was considered, but due to the need of GI bleeding scan (GIBS) prior to embolization, it was decided that an emergency surgery was a better approach. Subsequently, a joint operation was scheduled, involving a digestive surgeon and an obstetrician-gynecologist. During the surgery, active bleeding from the cancer mass was observed. Thus, a total abdominal hysterectomy with a bilateral salpingo-oophorectomy was conducted, continued with ileal resection at approximately 10 cm from the ileocecal valve. A double barrel stoma was also made during the surgery, and the patient was admitted to the ICU postoperatively. However, the patient subsequently developed sepsis, which ultimately led to the patient’s death. Subsequent pathological examination with samples taken during surgery and colonoscopy confirmed choriocarcinoma in this patient.

## Discussion

Choriocarcinoma is a rare but highly malignant form of GTD with a distinct epidemiological pattern where it is more commonly observed in Asian and African populations compared to Western countries [1,16]. The disease can arise following a molar pregnancy, normal pregnancy, miscarriage, or ectopic pregnancy, though the risk is highest in cases of complete hydatidiform mole. The incidence of choriocarcinoma varies, with reported rates ranging from 1 in 40,000 normal pregnancies to as high as 1 in 1,500 in cases following a complete molar pregnancy [17,18].

The most commonly affected sites for metastasis include the lungs (75-80% of cases), liver (10-20%), and brain (10-20%) [19-21]. Less frequent metastatic sites include the kidneys, spleen, and GI tract. In our case, the patient had a combination of intestinal, pulmonary, and liver metastasis. A retrospective study by Zong et al. showed that liver metastasis is a poor prognostic factor for choriocarcinoma [22]. Indeed, in FIGO classification, liver metastasis is one of the contributors to high score.

Currently, data on intestinal metastasis prevalence are not available. It is accepted that metastasis to the GI tract is exceedingly rare but carries significant clinical implications [12]. The small intestine is an uncommon site of involvement not just in choriocarcinoma but also in many cancers as well, and when it does occur, it typically presents with GI bleeding, bowel obstruction, or perforation. The rarity of such metastases can lead to delays in diagnosis, as symptoms are often attributed to more common GI conditions such as peptic ulcers, inflammatory bowel disease, or colorectal malignancies. In the present case, the patient experienced chronic hematochezia, which progressively worsened, ultimately leading to hemorrhagic shock and necessitating emergency intervention.

The pathophysiology of intestinal metastasis in choriocarcinoma remains poorly understood. One hypothesis is through hematogenous spread as the intestine is highly vascularized, which may increase the chances of cancer cell implantation [12]. Another probable mechanism includes implantation to the omentum and subsequent hematogenous spread to the intestinal tissue, as described by Wang et al. [12]. All in all, given the usual aggressive nature of choriocarcinoma, these metastatic lesions tend to invade deeply into the bowel wall, predisposing patients to complications such as ulceration, perforation, and massive hemorrhage.

Treatment of choriocarcinoma is primarily based on risk stratification using the World Health Organization (WHO) prognostic scoring system and the FIGO staging system [7,23,24]. Low-risk cases are treated successfully with single-agent chemotherapy, such as methotrexate or actinomycin D [7]. High-risk cases, particularly those with widespread metastases, require multi-agent chemotherapy, commonly EMA-CO (etoposide, methotrexate, actinomycin D, cyclophosphamide, and vincristine) [7]. According to the National Comprehensive Cancer Network (NCCN) clinical guidelines, treatment for metastatic choriocarcinoma includes etoposide, methotrexate, and dactinomycin alternating weekly with cyclophosphamide and vincristine [25].

Despite the aggressive nature of choriocarcinoma, intestinal metastases are often treatable if diagnosed in time. The role of colonoscopy and imaging modalities such as contrast-enhanced CT scans is paramount in identifying these rare metastatic sites. In this case, colonoscopy revealed an actively bleeding mass in the terminal ileum, which was later confirmed as choriocarcinoma on histopathology. The role of metastatic lesions biopsy in choriocarcinoma is still under investigation. In other cancers such as breast cancer, biopsies from metastatic lesions may give discordance results in hormonal receptor status [26]. Often, if the primary cancer is well established, a metastasized lesions biopsy may not be necessary. In our case, as the patient’s molar pregnancy had been previously operated, biopsy of the metastasized lesion is necessary to confirm diagnosis.

Surgical intervention remains a viable option in cases of GI metastasis, particularly when bleeding is refractory to conservative measures [7]. Another option is transcatheter arterial embolization (TAE) as a minimally invasive alternative approach for GI bleeding. As described in several case reports, surgical intervention or arterial embolization can be used to stop massive GI bleeding from choriocarcinoma [27-29].

According to a study by Lee et al., it is stated that TAE can be considered in lower GI bleeding patients where urgent colonoscopy cannot be performed or when hemodynamic resuscitation fails to improve vital signs [30]. A meta-analysis conducted by Tarasconi et al. that compared TAE with surgical approach to stop bleeding in non-variceal upper GI bleeding showed that TAE had a higher rebleeding rate, with similar mortality rates [31]. To the best of authors’ knowledge, there is currently no meta-analysis that compares TAE with surgical approach in lower GI bleeding. However, a study conducted in Finland showed that the complication rate of TAE in lower GI bleeding was 36%, with 13% of patients with complications requiring bowel resection [32]. A recent multicenter study by Hosse et al. demonstrated that TAE has a high clinical success rate; however, severe ischemia-related adverse events occurred in 14% of the study patients [33]. We believe that the high vasculature in the small intestines and colon may cause a risk of ischemia when conducting TAE in lower GI. Thus, a systematic review and meta-analysis that compares TAE with surgical and/or endoscopic approach in lower GI bleeding should be conducted.

Another important consideration when choosing TAE approach or not to stop profuse GI bleeding is its availability. TAE requires specialized equipment and experienced operators, which may make TAE not available in developing countries. Furthermore, the rapid availability of surgical approach may be preferred than TAE. In our case, due to high-volume cases of surgical approach for lower GI bleeding and lack of immediate access to TAE in our center, a surgical approach was chosen for the patient. Thus, the patient underwent a total abdominal hysterectomy with a bilateral salpingo-oophorectomy. A study that compared open total abdominal hysterectomy with minimally invasive surgery showed no difference in oncologic outcomes and bleeding outcomes [34]. However, patients with widespread metastases are at a high risk for postoperative complications, including sepsis, as seen in this case, which may contribute to mortality.

Chemotherapy may also be given in patients with lower GI bleeding [35,36]. In a case report by Kamel et al., in a patient with lower GI bleeding from choriocarcinoma metastasis, the bleeding was successfully stopped after the first cycle of chemotherapy, indicating the role of chemotherapy in stopping bleeding [35]. However, there is also a possibility that chemotherapy may aggravate the GI bleeding [37]. Regardless, in our case, chemotherapy was not given since the national insurance system of Indonesia only covers chemotherapy when pathological examinations’ data are available.

All in all, this case report highlights the challenges associated with managing late-stage choriocarcinoma that metastasizes to the small intestines and emphasizes the need for early surveillance in high-risk patients, particularly those with a history of molar pregnancy who did not receive adequate follow-up care. However, in developing countries such as Indonesia, this may not be possible due to inadequate health care system.

Based on the case, there are several avenues that could had been done to save this patient. Firstly, follow-up after the molar pregnancy should had been conducted to diagnose relapse early. However, this was not done by the other hospital due to insurance limitations. Secondly, the bleeding stopped through arterial embolization is likely to have a lower risk of sepsis, which may have prevented the death of this patient.

## Conclusions

In conclusion, while choriocarcinoma is a highly treatable malignancy with chemotherapy, its potential to metastasize to rare sites such as the intestine poses significant diagnostic and therapeutic challenges. Awareness of atypical presentations and the inclusion of GI metastases in the differential diagnosis of unexplained GI bleeding in patients with a history of molar pregnancy are important. In cases of lower GI bleeding due to choriocarcinoma metastasis, treatment options include chemotherapy, surgical intervention, and TAE. Additionally, post-surgical follow-up of molar pregnancy with serial hCG testing and imaging is crucial for the early detection of relapse.
